# Risk Factors for Single and Multiple Recurrences for Endoscopic Retrograde Cholangiopancreatography and Open Choledochotomy in Treating Choledocholithiasis

**DOI:** 10.1155/2023/4738985

**Published:** 2023-10-31

**Authors:** Yao Wu, Ying Zhang, Xiao Meng Jiang, Chen Jing Xu, Yan Yan Wang, Jin Yuan Gu, Yi Li, Shun Fu Xu

**Affiliations:** ^1^Sir Run Run Hospital, Nanjing Medical University, Nanjing 211100, China; ^2^School of Public Health, Southeast University, Nanjing 211189, China; ^3^Jiangsu Province Hospital, Nanjing Medical University, Nanjing 210029, China

## Abstract

**Background:**

There are few studies comparing recurrences between endoscopic retrograde cholangiopancreatography (ERCP) and open choledochotomy (OCT).

**Aims:**

To compare the effect of different surgical methods on single and multiple recurrences of choledocholithiasis.

**Methods:**

A total of 1255 patients with choledocholithiasis who underwent ERCP or OCT were retrospectively studied. The recurrence of choledocholithiasis was calculated by the Kaplan–Meier method with the log-rank test. Multivariate analyses of recurrent choledocholithiasis were performed by introducing variables with *P* < 0.20 in univariate analysis into the logistic regression model.

**Results:**

A total of 204 (16.7%, 204/1225) patients relapsed. Among the 204 patients, 74.5% relapsed within three years after surgery, of whom 39.7% (81/204) had multiple relapses (≥ 2). The recurrence rate of ERCP (17.2%, 119/692) was higher than that of OCT (15.1%, 85/563), but the difference was not statistically significant. The independent risk factors for a single recurrence of choledocholithiasis were diabetes, stone number ≥ 2, maximum stone diameter ≥ 15 mm, sedentary occupation, the approach of ERCP (EST or EPBD), periampullary diverticulum, primary suture, high-fat diet (postoperative), frequency of weekly vegetable intake (< 4, postoperative), and drinking (postoperative). However, the ERCP approach (EST or EPBD), OCT approach (LCBDE), primary suture, high-fat diet (postoperative), and frequency of weekly vegetable intake (< 4, postoperative) were independent risk factors for multiple recurrences of choledocholithiasis.

**Conclusion:**

Patients with choledocholithiasis should be followed up regularly for one to three years after treatment. Stone *number* ≥ 2, diabetes mellitus, periampullary diverticulum, surgical methods, and lifestyle are all risk factors for the recurrence of choledocholithiasis. ERCP is still the preferred surgical method based on the advantages of low risk of cholangitis recurrence, less hospital stay, minimally invasive surgery, fewer postoperative complications, and easier acceptance by elderly patients. In addition to optimizing the treatment plans, postoperative lifestyle management is also vital.

## 1. Introduction

Choledocholithiasis is a chronic and recurrent hepatobiliary disease whose pathological basis is impaired metabolism of cholesterol, bilirubin, and bile acids. As one of the common gastrointestinal diseases, choledocholithiasis poses a substantial economic burden to the health care system. The incidence of cholelithiasis is 5% to 15%, and the incidence of choledocholithiasis is approximately 5%-30% [1, 2]. In recent years, the incidence of choledocholithiasis has presented a rising trend as a result of changes in lifestyle and diet structure. In Western countries, gallstones are mostly discharged into and retained in the common bile duct, and approximately 15% of patients with cholecystolithiasis also have choledocholithiasis. In Asia, common bile duct stones are mostly plain brown stones or mixed stones [2].

The main treatment methods for choledocholithiasis include endoscopic retrograde cholangiopancreatography (ERCP), laparoscopic common bile duct exploration (LCBDE), open common bile duct exploration (OCBDE), and litholysis. ERCP is the most common treatment for choledocholithiasis. However, many follow-up observations have shown that the recurrence rate of choledocholithiasis after ERCP is 4%-25% [3]. It is generally believed that the most common time of recurrence of choledocholithiasis is around six months after the complete removal of the primary stone [4].

There are a large number of studies on risk factors for recurrences of choledocholithiasis. However, there is no consensus on the risk factors for postoperative recurrences of choledocholithiasis. The factors influencing the recurrences of choledocholithiasis are complex and cannot be explained by a single factor. They include factors related to first-episode stones, congenital factors, biological factors, behavioral intervention factors, and the number of stone recurrences [5].

In this study, we analyzed the risk factors for postoperative recurrences of choledocholithiasis with clinical data and follow-up data. Thus, our results provide practical advice for selecting a treatment plan and preventing recurrences.

## 2. Methods

### 2.1. Patients

Medical records of patients who underwent the initial treatment (ERPC or OCT) and follow-up checks for choledocholithiasis between January 2010 and December 2017 in the First Affiliated Hospital of Nanjing Medical University and the Sir Run Run Hospital affiliated with Nanjing Medical University were retrospectively reviewed. Patients were those operated for cholecystolithiasis and choledocholithiasis. Data obtained by regular telephone follow-up were also analyzed. The exclusion criteria were as follows: (i) combination of benign or malignant biliary stricture; (ii) permanent biliary stent implantation; (iii) complicated with intrahepatic bile duct stones; (iv) cirrhosis; and (v) incomplete medical or follow-up data.

Oral consent was obtained from the study participants.

**Figure 1:**
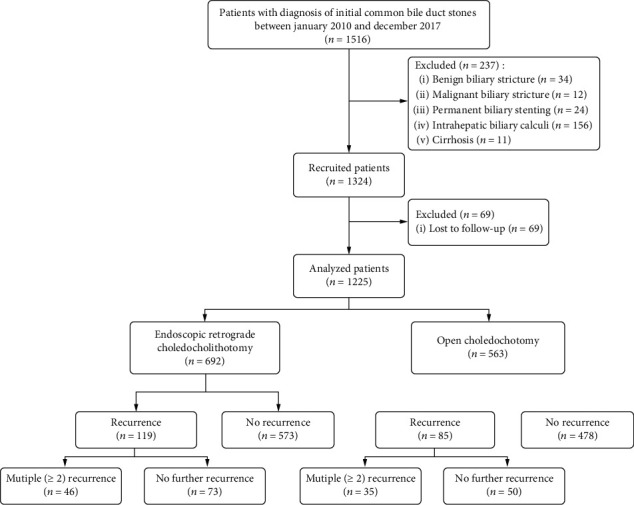
Study flowchart.

A total of 1561 patients who met the inclusion criteria were enrolled. After confirmation by imaging and/or pathological examination, 34 cases with benign biliary stricture, 12 cases with malignant biliary stricture, 24 cases with permanent biliary stenting, 156 cases with intrahepatic biliary calculi, and 11 cases with cirrhosis were excluded. Therefore, 237 patients were excluded, and 1324 patients met the inclusion criteria. Additionally, 69 patients were lost to follow-up. Finally, 1255 patients were enrolled for the analysis (Figure 1).

### 2.2. Procedures

All operations were performed by experienced endoscopists and surgeons. In addition, medical records and follow-up data were collected by professionals. The patient's medical records and laboratory and imaging findings were carefully evaluated before surgery. The surgery was performed under anesthesia after excluding contraindications.

### 2.3. Endoscopic Retrograde Cholangiopancreatography

ERCP was performed with a standard duodenoscope (JF-260V/TJP-260V; Olympus, Tokyo, Japan). The endoscope was successfully inserted into the descending segment of duodenum, and the common opening of the bile duct and pancreatic duct (duodenal papilla) was found. The contrast tube was inserted into the nipple, and contrast agent was injected. The distribution of contrast agent was viewed under the X-ray line, the filling defect was found, and the presence of choledocholithiasis was confirmed. Then, EST, EPBD, or EST+EPBD were performed to remove the stone with a stone net basket. This is usually performed by endoscopic cholangiopancreatography (ERCP)+endoscopic sphincterotomy (EST)+stone removal. Endoscopic papillary balloon dilatation (EBPD) may be an alternative to EST in patients with coagulation disorders, but the size of the calculi that can be obtained appears to be limited [6]. Therefore, EST combined with EPLBD (endoscopic papillary large-balloon dilation) is also used to treat choledocholithiasis. Endoscopic mechanical lithotripsy (EML) was used if necessary. Common bile duct retrograde angiography was performed again to ensure that there was no stone residue. A nasobiliary duct was implanted into the common bile duct.

### 2.4. Open Choledochotomy

Open choledochotomy was performed by LCBDE (laparoscopic common bile duct exploration) or OCBDE (open common bile duct exploration). The calculi were removed after the common bile duct was cut open, and then a primary suture or T-tube was placed. Cholangiography was performed with a T-tube 14-21 days after surgery. If there was no stone, the T-tube was clamped and removed one month later.

### 2.5. Follow-Up Procedures

Patients were followed up every three to six months after removing common bile duct stones. Clinical symptoms (fever, abdominal pain, jaundice, etc.), hematological tests, including liver function, and abdominal ultrasound were evaluated. Abdominal computed tomography (CT), magnetic resonance cholangiopancreatography (MRCP), and endoscopic ultrasonography or angiography were performed when choledocholithiasis was highly suspected. Hospitalization was recommended when further examination and/or treatment are deemed necessary.

Recurrence of choledocholithiasis was defined as the occurrence of choledocholithiasis six months after the first complete choledocholithiasis removal. The presence of choledocholithiasis was confirmed by one of the following three methods: abdominal computed tomography, magnetic resonance cholangiopancreatography, and endoscopic ultrasonography or angiography.

### 2.6. Statistical Analysis

SPSS version 23.0 (IBM, Armonk, NY, USA) was used for data analysis. The recurrence of choledocholithiasis was calculated by the Kaplan–Meier method with the log-rank test. Multivariate analyses of recurrent choledocholithiasis were performed by introducing variables with *P* < 0.20 in the univariate analysis into the logistic regression model. *P* < 0.05 was considered statistically significant.

## 3. Results

A total of 1255 patients with choledocholithiasis were included in this retrospective analysis. The follow-up period after the initial treatment of choledocholithiasis was 5.0 ± 1.9 years. The mean patient age was 59.1 ± 15.4 years, and 49.8% of the patients were women. A total of 204 (16.7%, 204/1225) patients relapsed, including 123 patients with one recurrence, 50 patients with two recurrences, 18 patients with three recurrences, and 13 patients with four or more recurrences. Meanwhile, among the 204 patients who relapsed, 39 (19.1%, 39/204) relapsed in the first year after surgery, 62 (30.4%, 62/204) in the second year, 51 (25%, 51/204) in the third year, 27 (13.2%, 27/204) in the fourth year, and 25 (12.3%, 25/204) in the fifth to the eighth years.

Of the 1255 patients, 692 were treated with ERCP and 563 with OCT. The recurrence rate after ERCP was 17.2% (119/692), and the recurrence rate after OCT was 15.1% (85/563).

### 3.1. Analysis of Risk Factors for Single Recurrence of Choledocholithiasis

**Figure 2:**
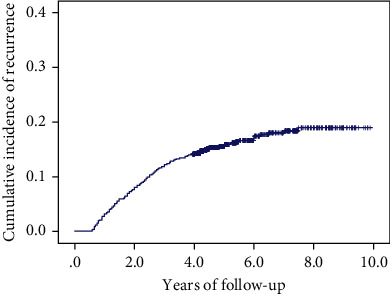
The cumulative incidence of recurrences of choledocholithiasis.

The cumulative incidence of recurrence of choledocholithiasis is shown in Figure 2. All patient characteristics of those with and without common bile duct stone recurrence and the analysis (univariate and multivariate analyses) are shown in Table 1. In the multivariate analysis, diabetes (OR = 2.14; 95% CI: 1.197-3.828; *p* = 0.010), stone number ≥ 2 (OR = 3.538; 95% CI: 2.373-5.276; *p* < 0.001), maximum stone diameter ≥ 15 mm (OR = 4.322; 95% CI: 3.056-6.114; *p* < 0.001), sedentary occupation (OR = 2.357; 95% CI: 1.666-2.334; *p* < 0.001), high-fat diet (postoperative) (OR = 3.496; 95% CI: 1.939-6.304; *p* < 0.001), frequency of weekly vegetable intake (< 4, postoperative) (OR = 6.075; 95% CI: 2.981-12.379; *p* < 0.001), and drinking (postoperative) (OR = 2.125; 95% CI: 1.320-3.420; *p* = 0.002) were independent risk factors for recurrence of choledocholithiasis.

The characteristics of patients with ERCP and the analysis (univariate and multivariate analyses) are shown in Table 2. In the multivariate analysis, stone number ≥ 2 (OR = 2.818; 95% CI: 1.684-4.715; *p* < 0.001), maximum stone diameter ≥ 15 mm (OR = 2.954; 95% CI: 1.812-4.816; *p* < 0.001), approach of ERCP (EST or EPBD) (OR = 2.519; 95% CI: 1.547-4.102; *p* < 0.001), periampullary diverticulum (OR = 2.099; 95% CI: 1.293-3.409; *p* = 0.003), sedentary occupation (OR = 1.796; 95% CI: 1.145-2.819; *p* = 0.011), frequency of weekly vegetable intake (< 4, postoperative) (OR = 13.898; 95% CI: 5.327-36.259; *p* < 0.001), and drinking (postoperative) (OR = 2.109; 95% CI: 1.184-3.757; *p* = 0.011) were independent risk factors for recurrence of choledocholithiasis. Periampullary duodenal diverticulum (PAD) was found in 140 cases (20.2%, 140/692), including type I (10%, 14/140), type II (28.6%, 40/140), and type III (61.4%, 86/140). The recurrence rate of type III was 15.1% (13/86), while that of common bile duct stones in type I+II was as high as 40.8% (22/54).

The characteristics of the patients with OCT and the analysis (univariate and multivariate analyses) are shown in Table 3. In the multivariate analysis, age ≥ 65 years (OR = 2.242; 95% CI: 1.188-4.234; *p* = 0.013), stone number ≥ 2 (OR = 7.101; 95% CI: 2.334-14.742; *p* < 0.001), maximum stone diameter ≥ 15 mm (OR = 15.837; 95% CI: 8.050-31.158; *p* < 0.001), primary suture (OR = 2.846; 95% CI: 1.194-6.785; *p* = 0.018), sedentary occupation (OR = 4.385; 95% CI: 2.355-8.162; *p* < 0.001), high-fat diet (postoperative) (OR = 3.699; 95% CI: 1.337-10.233; *p* = 0.012), frequency of weekly vegetable intake (< 4, postoperative) (OR = 4.153; 95% CI: 1.192-14.469; *p* = 0.025), and drinking (postoperative) (OR = 3.162; 95% CI: 1.270-7.872; *p* = 0.013) were independent risk factors for recurrence.

### 3.2. Analysis of Risk Factors for Multiple Recurrences of Choledocholithiasis

**Figure 3:**
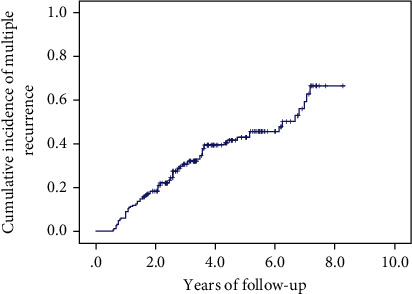
The cumulative incidence of multiple recurrences of choledocholithiasis.

We compared multiple recurrent choledocholithiasis with single recurrence. The cumulative incidence of multiple recurrences for choledocholithiasis is shown in Figure 3. All patient characteristics of those with and without common bile duct stones with multiple recurrences and analysis (univariate and multivariate analysis) are shown in Table 4. In the multivariate analysis, a high-fat diet (postoperative) (OR = 4.625; 95% CI: 2.177-9.828; *p* < 0.001) was the only independent risk factor for multiple recurrences of choledocholithiasis.

The characteristics of the patients with ERCP and the analysis (univariate and multivariate analyses) are shown in Table 5. In the multivariate analysis, the ERCP approach (EST or EPBD) (OR = 4.865 95% CI: 1.814-13.050; *p* = 0.002) and frequency of weekly vegetable intake (< 4, postoperative) (OR = 12.819; 95% CI: 3.495-47.081; *p* < 0.001) were independent risk factors for multiple recurrences of choledocholithiasis.

The characteristics of the patients with OCT and the analysis (univariate and multivariate analyses) are shown in Table 6. In the multivariate analysis, the OCT (LCBDE) approach (OR = 5.162; 95% CI: 1.649-16.157; *p* = 0.005), primary suture (OR = 7.493; 95% CI: 1.814-30.964; *p* = 0.005), and frequency of weekly vegetable intake (< 4, postoperative) (OR = 4.175; 95% CI: 1.013-17.206; *p* = 0.048) were independent risk factors for multiple recurrences of choledocholithiasis.

A total of 74 patients with common choledocholithiasis combined with cholangitis were found before the operation, of which 7 of 45 patients with ERCP and 8 of 29 patients with OCT had recurrent cholangitis. Therefore, the recurrence rate of cholangitis via ERCP was 15.6%, and the recurrence rate of cholangitis via OCT was 27.6%.

The average length of hospital stay was 3.3 days after ERCP, 4.9 days after LCBDE, and 5.6 days after OCBDE. The total treatment cost of ERCP is approximately 3201.63$, LCBDE is approximately 3053.77$, and OCBDE is approximately 3018.80$. After ERCP, 14 cases developed mild pancreatitis, 1 case developed moderate pancreatitis, 7 cases developed hyperamylasemia, and 3 cases developed cholangitis. No gastrointestinal bleeding, perforation, death, or other complications occurred. Biliary fistula occurred in 1 case, and cholangitis occurred in 4 cases after LCBDE. Three patients developed cholangitis after OCBDE. Twenty-one patients with OCT lithotomy complained of incision pain and unexplained abdominal discomfort after discharge.

## 4. Discussion

This study showed that the recurrence rate of choledocholithiasis was 16.7%. Of all 204 patients with an initial recurrence, 81 (39.7%) had multiple (≥ 2) relapses. Meanwhile, among the 204 patients who relapsed, 39 (19.1%, 39/204) relapsed in the first year after surgery, 62 (30.4%, 62/204) in the second year, 51 (25%, 51/204) in the third year, 27 (13.2%, 27/204) in the fourth year, and 25 (12.3%, 25/204) in the fifth to eighth years. A total of 74.4% of patients with choledocholithiasis had recurrences within three years after surgery. The recurrence rate of patients with a first recurrence was not low. Therefore, regular follow-up within one to three years after surgery is beneficial.

Women are more prone to choledocholithiasis due to high estrogen levels, lack of exercise, and so on. The bile duct contraction force is decreased, resulting in easy bile deposition and precipitate crystallization. However, this study and most other studies showed no significant correlation between sex and choledocholithiasis recurrence [7]. This suggests that gender may only be a risk factor for the first occurrence of choledocholithiasis rather than recurrences.

It has been reported that one-third of patients with recurrent choledocholithiasis are over 65 years old [5]. We found that older age (elderly patients aged 65 years or older) was a risk factor for the recurrence of choledocholithiasis after OCT. The functional recovery of bile ducts after open choledochotomy is not as good in older patients as in younger patients. ERCP is recommended for elderly (≥ 65 years old) patients with choledocholithiasis, which has the advantages of minor trauma and faster recovery.

It has been reported that the ABCG5/8 (encoding the hepatobiliary cholesterol transporter 5/8) allele is related to the recurrence of choledocholithiasis, and the variant gene ABCG D19H is currently recognized as a genetic risk factor for the formation of gallstones, which can effectively predict the recurrence of choledocholithiasis [8]. However, this study found no significant correlation between a family history of biliary stones and common bile duct stone recurrence, which may be associated with patient recall bias, and a larger sample size is still needed.

Patients with diabetes are more likely to have recurrent choledocholithiasis, so blood glucose control is significant for patients with both diabetes and choledocholithiasis.

This study showed that patients with ≥ 2 stones and a maximum stone diameter ≥ 15 mm were more likely to have recurrent choledocholithiasis. This is consistent with the findings of Li and Song [4, 9]. The larger the stone diameter is, the greater the influence on the normal motor function of the bile duct. The Oddi sphincter function is easily affected. At the same time, most large stones require lithotripsy. Silt stones are more likely to remain in the bile duct after endoscopic treatment, and the small stone fragments missed by cholangiography may lead to stone aggregation and recurrence.

Some clinicians believe that the recurrence rate of choledocholithiasis is higher after ERCP than after OCT, which may be associated with the occurrence of pancreatitis. However, there are scarce data on whether there is a difference between the two methods for treating choledocholithiasis. Our study found that the recurrence rate of choledocholithiasis treated by ERCP was higher than that treated by OCT, but the difference was not statistically significant. The ERCP approach (EST or EPBD) was an independent risk factor for both single and multiple recurrences of choledocholithiasis. It is worth mentioning that EST+EPLBD during ERCP may be a “protective factor” for the recurrence of choledocholithiasis. The function of the Oddi sphincter is significantly damaged by EST alone, and the duodenal fluid easily flows back into the biliary tract, resulting in postoperative biliary tract infection and long-term recurrence of calculi. EPBD preserves 70% of the Oddi sphincter function, but EBPD alone seems to limit the calculus size that can be removed. EST+EPBD causes less damage to the Oddi sphincter due to its small incision.

Compared with EST alone, there was no significant difference in the success rate of small incision EST combined with EPBD in treating common bile duct stones [10]. However, studies have shown that lithotripsy is an independent risk factor for recurrent choledocholithiasis [11]. Our multivariate analysis did not reach this conclusion due to the full effect of nasobiliary drainage.

For patients treated with OCT, although the difference in approach (LCBDE or OCBDE) was not statistically significant for recurrence, we found that the OCT approach (LCBDE) was an independent risk factor for multiple recurrences of choledocholithiasis. Primary suture was an independent risk factor for both single and multiple recurrences of choledocholithiasis. We believe that OCBDE can expose the field of vision more thoroughly for lithotomy, and T-tube drainage can assist bile drainage to recover better bile duct function. The main purpose of indwelling T-tube after surgery is to effectively drain bile, thereby reducing the pressure of the biliary tract and avoiding postoperative biliary tract stenosis. Therefore, it can reduce the risk of recurrence for choledocholithiasis. Although the operation time of primary suture patients is short, bile leakage and other related complications are easy to occur. This aggravates the patient's pain while causing biliary tract damage, more prone to the recurrence of choledocholithiasis.

The 2017 UK guidelines for treating choledocholithiasis stated that all patients with cholecystolithiasis or gallstones should undergo cholecystectomy unless there are specific cases where surgery is not permitted [12]. However, Yoo et al. [13] showed that a prior history of cholecystectomy could increase the risk of recurrence of choledocholithiasis after LCBDE. The gallbladder can excrete bile and flush the biliary tract to prevent stone formation. This function is lost when the gallbladder is removed. In this study, cholecystectomy was defined as cholecystectomy for patients with choledocholithiasis combined with cholecystolithiasis within one week after ERCP or simultaneous OCT with cholecystectomy. This study showed that cholecystectomy is not a risk factor for recurrent choledocholithiasis. It may have something to do with the timing of gallbladder removal, so further studies with larger sample sizes are needed to confirm this.

Most of the periampullary duodenal diverticula (PAD) are located within 2-3 cm of the duodenal nipple. PAD can be divided into three types: type I: the nipple is located within the diverticulum; type II: the nipple is located at the inner edge of the diverticulum; and type III: the nipple is located outside the diverticulum. This study showed that PAD is a risk factor for the recurrence of choledocholithiasis. The mechanism by which PAD causes recurrence is still unclear. The possible mechanism is as follows: (i) the bacteria overgrow and diffuse into the biliary system, while food deposition in the diverticulum forms a suitable culture medium for bacteria. The bacteria collected from the diverticulum belong to the same strain as those isolated from the biliary tract. The most common bacteria included *Escherichia coli*, *Streptococcus faecalis*, *Proteus*, *Klebsiella*, *Pseudomonas aeruginosa*, and anaerobic bacteria. (ii) Compared with patients without PAD, the activity of *β*-glucuronate in bile is increased in patients with PAD, which promotes bilirubin stone formation. (iii) Diverticulum around the ampullary may affect the normal anatomy of the nipple, compress the distal common bile duct and dilate the bile duct, interfere with the excretion of bile and pancreatic fluid, and promote the formation of calculi. Patients with a large diverticulum (>2 cm) are more likely to form stones. (iv) Oddi's sphincter dysfunction often occurs in the diverticulum around the ampulla. Increased biliary tract pressure or biliary tract spasm leads to blocked bile flow, which may increase the recurrence rate of common bile duct stones [14].

We cannot predict the occurrence of many diseases in advance, but we can do an excellent job of postdisease life management. We found that sedentary occupation, high-fat diet (postoperative), frequency of weekly vegetable intake (< 4, postoperative), and drinking (postoperative) were independent risk factors for recurrences of choledocholithiasis. Patients with sedentary occupations are more likely to relapse, which may be related to lack of exercise, poor metabolism, and poor biliary muscle function. Abstaining from alcohol was defined as less than 8 liters of white wine, 330 ml of beer, or 120 ml of wine per month.

The incidence of recurrent cholangitis in patients with choledocholithiasis combined with cholangitis after ERCP (15.6%) is lower than that of patients with OCT (27.6%). The length of hospital stay after ERCP (3.3 d) was shorter than that after OCT (LCBDE: 4.9 d; OCBDE: 5.6 d), although the total cost was slightly higher than that after OCT. The incidence of complications after ERCP was 3.6%, and all patients recovered with conservative management. Although the incidence of complications after OCT was lower (1.4%), 3.7% of patients complained of postoperative incision pain and prolonged unexplained abdominal discomfort.

In conclusion, the recurrence rate of patients with an initial recurrence was significantly high. It is suggested that patients with choledocholithiasis should be followed up regularly for one to three years after treatment. Quite a few clinicians and researchers believed that the recurrence rate of choledocholithiasis after OCT was lower than that after ERCP, and our study also confirmed this, but there was no statistical difference. Stone number ≥ 2, maximum stone diameter ≥ 15 mm, diabetes mellitus, periampullary diverticulum, surgical methods, and lifestyle are all risk factors for the recurrence of choledocholithiasis. ERCP is still the preferred surgical method based on the advantages of low risk of cholangitis recurrence, less hospital stay, minimally invasive surgery, fewer postoperative complications, and easier acceptance by elderly patients. The approach of ERCP (EST or EPBD) was an independent risk factor for both single and multiple recurrences of choledocholithiasis. Hence, EST+EPBD is recommended as the preferred surgical method for choledocholithiasis. In addition to optimizing the treatment plans, postoperative lifestyle management is also vital for preventing common bile duct stones.

## Figures and Tables

**Table 1:** Risk factors for common bile duct stone recurrence (all analyzed patients).

	Recurrence (*n* = 204)	Nonrecurrence (*n* = 1051)	Univariate analysis	Multivariate analysis		
			*P*	OR	95% CI	*P*
Sex						
Male	99	511	0.973	—	—	—
Female	105	540				
Age (years)						
<65	119	790	<0.001	—	—	—
≥65	85	261				
Diabetes						
No	181	992	0.002	2.14	1.197-3.828	0.010
Yes	23	59				
Family history of biliary stones						
No	156	850	0.177	—	—	—
Yes	48	201				
Stone number						
Single	48	471	<0.001	3.538	2.373-5.276	<0.001
Multiple	156	580				
Maximum stone diameter (mm)						
<15	68	715	<0.001	4.322	3.056-6.114	<0.001
≥15	136	336				
Operation method						
Open choledochotomy	85	478	0.222	—	—	—
Endoscopic retrograde choledocholithotomy	119	573				
Cholecystectomy						
No	153	837	0.15	—	—	—
Yes	51	214				
Sedentary occupations						
No	74	617	<0.001	2.357	1.666-2.334	<0.001
Yes	130	434				
High-fat diet (postoperative)						
No	165	1004	<0.001	3.496	1.939-6.304	<0.001
Yes	39	47				
Frequency of weekly vegetable intake (postoperative)						
≥4	173	1030	<0.001	6.075	2.981-12.379	<0.001
<4	31	21				
Drinking (postoperative)						
No	165	951	<0.001	2.125	1.320-3.420	0.002
Yes	39	100				
Smoking (postoperative)						
No	180	989	0.002	—	—	—
Yes	24	62				

**Table 2:** Risk factors for common bile duct stone recurrence (patients with ERCP).

	Recurrence (*n* = 119)	Nonrecurrence (*n* = 573)	Univariate analysis	Multivariate analysis		
			*P*	OR	95% CI	*P*
Sex						
Male	55	285	0.572	—	—	—
Female	64	288				
Age (years)						
<65	71	420	0.003	—	—	—
≥65	48	153				
Diabetes						
No	106	538	0.039	—	—	—
Yes	13	35				
Family history of biliary stones						
No	90	453	0.382	—	—	—
Yes	29	120				
Stone number						
Single	23	195	0.002	2.818	1.684-4.715	<0.001
Multiple	67	325				
Maximum stone diameter (mm)						
<15	37	291	<0.001	2.954	1.812-4.816	<0.001
≥15	53	162				
Endoscopic retrograde choledocholithotomy						
EST+EPLBD	37	210	0.049	2.519	1.547-4.102	<0.001
EST or EPBD	53	243				
Lithotripsy						
No	102	528	0.018	—	—	—
Yes	17	45				
Periampullary duodenal diverticulum						
No	79	473	<0.001	2.099	1.293-3.409	0.003
Yes	40	100				
Cholecystectomy						
No	86	435	0.422	—	—	—
Yes	33	138				
Sedentary occupations						
No	44	321	<0.001	1.796	1.145-2.819	0.011
Yes	75	252				
High-fat diet (postoperative)						
No	99	546	<0.001	—	—	—
Yes	20	27				
Frequency of weekly vegetable intake (postoperative)						
≥4	102	564	<0.001	13.898	5.327-36.259	<0.001
<4	17	9				
Drinking (postoperative)						
No	95	512	0.002	2.109	1.184-3.757	0.011
Yes	24	61				
Smoking (postoperative)						
No	108	542	0.09	—	—	—
Yes	11	31				

**Table 3:** Risk factors for common bile duct stone recurrence (patient with OCT).

	Recurrence (*n* = 85)	Nonrecurrence (*n* = 478)	Univariate analysis	Multivariate analysis		
			*P*	OR	95% CI	*P*
Sex						
Male	44	226	0.473	—	—	—
Female	41	252				
Age (years)						
<65	48	370	<0.001	2.242	1.188-4.234	0.013
≥65	37	108				
Diabetes						
No	74	454	0.017	—	—	—
Yes	10	24				
Family history of biliary stones						
No	66	397	0.316	—	—	—
Yes	19	81				
Stone number						
Single	15	222	<0.001	7.010	2.334-14.742	<0.001
Multiple	70	256				
Maximum stone diameter (mm)						
<15	20	349	<0.001	15.837	8.050-31.158	<0.001
≥15	65	129				
Open choledochotomy						
OCBDE	34	226	0.245	—	—	—
LCBDE	51	252				
T-tube drainage or primary suture						
T-tube drainage	69	426	0.044	2.846	1.194-6.785	0.018
Primary suture	16	52				
Cholecystectomy						
No	67	402	0.255	—	—	—
Yes	18	76				
Sedentary occupations						
No	30	296	<0.001	4.385	2.355-8.162	<0.001
Yes	55	182				
High-fat diet (postoperative)						
No	66	458	<0.001	3.699	1.337-10.233	0.012
Yes	19	20				
Frequency of weekly vegetable intake (postoperative)						
≥4	71	466	<0.001	4.153	1.192-14.469	0.025
<4	14	12				
Drinking (postoperative)						
No	70	439	0.008	3.162	1.270-7.872	0.013
Yes	15	39				
Smoking (postoperative)						
No	72	447	0.004	—	—	—
Yes	13	31				

**Table 4:** Risk factors for multiple common bile duct stone recurrences (all analyzed patients).

	Multiple recurrences (*n* = 81)	No further recurrence (*n* = 123)	Univariate analysis	Multivariate analysis		
			*P*	OR	95% CI	*P*
Sex						
Male	41	58	0.837	—	—	—
Female	40	65				
Age (years)						
<65	46	73	0.646	—	—	—
≥65	35	50				
Diabetes						
No	71	110	0.760	—	—	—
Yes	10	13				
Family history of biliary stones						
No	62	94	0.983	—	—	—
Yes	19	29				
Stone number						
Single	11	22	0.449	—	—	—
Multiple	51	72				
Maximum stone diameter(mm)						
<15	20	36	0.295	—	—	—
≥15	42	58				
Operation method						
Open choledochotomy	35	50	0.339	—	—	—
Endoscopic retrograde choledocholithotomy	46	73				
Cholecystectomy						
No	62	91	0.763	—	—	—
Yes	19	32				
Sedentary occupations						
No	34	40	0.223	—	—	—
Yes	47	83				
High-fat diet (postoperative)						
No	54	111	<0.001	4.625	2.177-9.828	<0.001
Yes	27	12				
Frequency of weekly vegetable intake (postoperative)						
≥4	59	114	0.001	—	—	—
<4	22	9				
Drinking (postoperative)						
No	62	103	0.408	—	—	—
Yes	19	20				
Smoking (postoperative)						
No	65	115	0.022	—	—	—
Yes	16	8				

**Table 5:** Risk factors for multiple common bile duct stone recurrences (patients with ERCP).

	Multiple recurrences (*n* = 46)	No further recurrence (*n* = 73)	Univariate analysis	Multivariate analysis		
			*P*	OR	95% CI	*P*
Sex						
Male	20	35	0.691	—	—	—
Female	26	38				
Age (years)						
<65	25	46	0.373	—	—	—
≥65	21	27				
Diabetes						
No	41	65	0.847	—	—	—
Yes	5	8				
Family history of biliary stones						
No	34	56	0.970	—	—	—
Yes	12	17				
Stone number						
Single	12	21	0.853	—	—	—
Multiple	21	52				
Maximum stone diameter (mm)						
<15	14	34	0.138	—	—	—
≥15	32	39				
Endoscopic retrograde choledocholithotomy						
EST+EPLBD	10	36	0.003	4.865	1.814-13.050	0.002
EST or EPBD	36	37				
Lithotripsy						
No	40	62	0.580	—	—	—
Yes	6	11				
Periampullary duodenal diverticulum						
No	31	48	0.509	—	—	—
Yes	15	25				
Cholecystectomy						
No	35	51	0.784	—	—	—
Yes	11	22				
Sedentary occupation						
No	17	27	0.986	—	—	—
Yes	29	46				
High-fat diet (postoperative)						
No	30	69	<0.001	—	—	—
Yes	16	4				
Frequency of weekly vegetable intake (postoperative)						
≥4	34	68	0.008	12.819	3.495-47.081	<0.001
<4	12	5				
Drinking (postoperative)						
No	34	61	0.139	—	—	—
Yes	12	12				
Smoking (postoperative)						
No	38	70	0.015	—	—	—
Yes	8	3				

**Table 6:** Risk factors for multiple common bile duct stone recurrences (patient with OCT).

	Multiple recurrences (*n* = 35)	No further recurrence (*n* = 50)	Univariate analysis	Multivariate analysis		
			*P*	OR	95% CI	*P*
Sex						
Male	21	23	0.317	—	—	—
Female	14	27				
Age (years)						
<65	21	27	0.919	—	—	—
≥65	14	23				
Diabetes						
No	30	45	0.781	—	—	—
Yes	5	5				
Family history of biliary stones						
No	28	38	0.892	—	—	—
Yes	7	12				
Stone number						
Single	5	10	0.292	—	—	—
Multiple	30	40				
Maximum stone diameter (mm)						
<15	9	11	0.952	—	—	—
≥15	26	39				
Open choledochotomy						
OCBDE	8	26	0.013	5.162	1.649-16.157	0.005
LCBDE	27	24				
T-tube drainage or primary suture						
T-tube drainage	23	46	0.023	7.493	1.814-30.964	0.005
Primary suture	12	4				
Cholecystectomy						
No	27	40	0.930	—	—	—
Yes	8	10				
Sedentary occupations						
No	18	37	0.067	—	—	—
Yes	17	13				
High-fat diet (postoperative)						
No	24	42	0.127	—	—	—
Yes	11	8				
Frequency of weekly vegetable intake (postoperative)						
≥4	25	46	0.016	4.175	1.013-17.206	0.048
<4	10	4				
Drinking (postoperative)						
No	28	42	0.667	—	—	—
Yes	7	8				
Smoking (postoperative)						
No	27	45	0.251	—	—	—
Yes	8	5				

## Data Availability

No underlying data was collected or produced in this study.
